# Perceived Crowding and Risk Perception According to Leisure Activity Type during COVID-19 Using Spatial Proximity

**DOI:** 10.3390/ijerph18020457

**Published:** 2021-01-08

**Authors:** Young-Jae Kim, Seung-Woo Kang

**Affiliations:** Department of Physical Education, Chung-Ang University, Seoul 06974, Korea; yjkim@cau.ac.kr

**Keywords:** COVID-19, perceived crowding, risk perception, spatial proximity, leisure activities, social distancing

## Abstract

This study aimed to investigate the difference in people’s perceived crowding and risk perception during leisure activities using the criteria of spatial proximity during the COVID-19 pandemic. COVID-19 is a viral respiratory tract disease that poses an increasing risk of infection through person-to-person transmission in a confined space or close proximity to an infected person. It is thus crucial to maintain a sufficiently safe distance from others during leisure activities. In this study, measures concerning leisure activity spaces and the current status of leisure activities were investigated. Data were gathered from a total of 1078 participants via an online survey conducted from 26 to 29 October 2020. Frequency analysis was performed to investigate the sample characteristics and exploratory factor analysis was performed to analyze the validity of the measurement tools. Results revealed that people’s perceived crowding of leisure activity spaces directly influenced their participation in leisure activities. Regarding age, those in their 20s were more aware of congestion and their risk perception was higher than those in their 40s and 50s. It was found that people perceived cultural and artistic activities to be dangerous as they often take place as part of tourism and leisure activities and amidst crowds. However, their high-risk perception indirectly influenced their participation patterns, making it difficult to enjoy leisure activities. To lower the risk perception of leisure activity spaces, it was necessary to secure more safe distancing than current regulations require. Future research must conduct a longitudinal investigation by objectively stratifying the degree of perceived crowding.

## 1. Introduction

The Korean government’s efforts to reduce the spread of Corona Virus Disease-19 (COVID-19) included social distancing. However, the adverse effects of social distancing manifested in the form of loneliness, depression, anxiety, and decreased physical activity among citizens. The government further imposed restrictions on multi-use facilities related to leisure activities, including group sports in indoor sports facilities [1]. The gathering of small groups was also restricted, and people were encouraged to return home early. The significance of the social distancing introduced to curtail the spread of COVID-19 was weakened by people’s perception that it restricted their activities by forcing them to maintain social distance in leisure activity spaces. From a sociological perspective, the crisis related to leisure activity spaces has had a significant impact [2]. Untact (a Korean shorthand expression for non-face-to-face contact) services are being implemented through changes in leisure activities, and the trend is shifting toward private leisure activities. That is, an increasing number of people are prioritizing leisure activities that can be enjoyed within the scope of “distancing in daily life”, such as camping or jogging. This reduces the risk of coming into contact with others. It further explains the popularity of small-group leisure activities in response to COVID-19 [3].

The pandemic inevitably caused the closure of facilities such as gyms, parks, playgrounds, outdoor play zones, and schools [4]. Even as outdoor activities are prioritized over indoor activities in multi-use facilities that are more vulnerable to the spread of viruses, outdoor leisure activities remain restricted [5,6]. Thus, people are attracted to partially permitted outdoor leisure spaces and tourist destinations and flock to these spaces as they did prior to the pandemic. Therefore, the number of restrictions to adhere to during leisure activities is increasing [7,8]. The increasing number of participants in leisure activities can cause the phenomenon of “crowding” [9], which is defined as a negative affective reaction to high-density settings [10]. Specifically, “perceived crowding” is the subjective and negative feeling of being restrained due to excessive external environmental stimuli [11,12], and refers to the psychological state of people occurring in specific spatial settings [13]. Moreover, crowding is distinct from density, which is an objective measure of the number of people per unit area (1 m^3^) [14]. It is divided into human crowding and spatial crowding [15,16,17].

Recent studies have reported that crowding perception is affected by psychological and environmental factors, rather than by visitor density. Eom and Han [18] reported that users of outdoor recreational facilities perceived crowding due to sociopsychological influences instead of spatial density. According to a study by Choon [19], experiences of leisure activities influence satisfaction with crowded settings, which is more affected by the surroundings than by spatial crowding. Non-spatial dimensions of proximity can serve as criteria for perceived crowding [20]. Additionally, it has been argued that while proximity is generally considered to be essential in our daily lives, excessive proximity can be harmful [21]. It was also found that while social proximity is crucial regarding a personal sense of responsibility and emotional intimacy, familiarity is less important in terms of privacy [22]. Stating that “space is special”, Longley, Goodchild, Maguire, and Rhind [23] highlighted the usefulness of an alternative research method modeling spatial dependence and spatial heterogeneity in explaining spatial effects in the leisure sector [20]. That is, spatial crowding has a direct effect on people’s lives, and people require social distances, or psychologically safe distances. This refers to the minimum distance that can fend off biological stress without being physically conspicuous, and individuals become anxious or stressed when unfamiliar persons or objects encroach upon this social distance [24]. Kennedy et al. demonstrated that humans show strong biological responses in the amygdala to personal space violation [25]. Consistently, a study noted that individual pedestrians have different genetic makeups and their needs for social distance vary according to their genetic type [26]. Accordant with changes over time, such as urbanization and intensified individualism in leisure activity spaces, there is a growing interest in the “third space”. This refers to a zone that can be occupied regularly by a person or a group of people to feel relaxed in public spaces [27]. Public spaces such as plazas, cafes, restaurants, parks, libraries, dormitories, museums, and exhibition halls are typical leisure activity spaces.

In the context of the pandemic, leisure activity spaces may have elements that cause discomfort such as noise and the gaze of others due to crowdedness within a limited space. However, leisure activities or outdoor physical activities are important for maintaining physical health and psychological stability, especially during a pandemic that causes psychological uncertainty. Nevertheless, as the external or environmental risk factors increase [28,29,30], personal spaces where anyone can enter are increasing and so are leisure activities in such spaces. These changes in the patterns of leisure activities further have a significant impact on public institutions. As the importance of leisure activity spaces grows, there is a need for research to standardize the concept of space [31].

Therefore, this study aimed to investigate the difference in perceived crowding and risk perception in leisure activity spaces during the pandemic using proxemic criteria. The results of this study may provide baseline data for proxemic information related to leisure activities during mass outbreaks of respiratory infectious diseases such as COVID-19.

## 2. Materials and Methods

### 2.1. Sample and Participants

The target population for this study consisted of adults residing in Korea, whether in rural or urban areas. An online questionnaire survey firm (Panel Now: https://www.panelnow.co.kr/) conducted an online survey from 26 to 29 October 2020. For the purposes of this study, we gathered information on each leisure activity participant’s perceived crowding and risk perception since January 2020, when the first case of COVID-19 was confirmed in Korea. Through multi-stage cluster sampling, 1200 questionnaires were collected from a total of 1300 distributed questionnaires, with a recovery rate of 92.3%. After excluding 122 questionnaires containing multiple responses or omissions, a total of 1078 respondents were included in the final analysis. The sample sizes were classified as 100 = poor; 200 = moderate, which is the sample size used for each grade in Comrey and Lee’s quantitative study [32]—300 = good; 500 = very good; and ≥1000 = excellent. The study was approved by the Institutional Review Board of Chung-Ang University (IRB1041078-202008-HRSB-218-01) and was conducted in compliance with the regulations of the Declaration of Helsinki, 1975. All participants consented to participate in the survey prior to conducting the study.

### 2.2. Measurements

The questionnaire, comprising perceived crowding, risk perception, spatial proximity, demographic characteristics, and leisure activity types, was administered as a cross-sectional survey. Based on previous international studies, each item was rated on a five-point Likert scale (1 = not at all likely; 2 = not likely; 3 = neutral; 4 = likely; 5 = very likely). Participants’ sociodemographic characteristics included gender, age, marital status, spatial proximity type, and leisure activity type. To assess the crowding and risk factors that may emerge during leisure activities, two items pertaining to perceived crowding and four items pertaining to risk perception were included. To establish the face validity of the questionnaire scale, the questionnaire was constructed in consultation with a three-member expert panel (one sociology expert and two leisure experts).

#### 2.2.1. Perceived Crowding

To assess perceived crowding, we designed a questionnaire by modifying and complementing the item for perceived crowding in the questionnaire developed by Heberlein and Vaske [33] and the item for expected use-intensity proposed by Graefe and Felder [34] and Hall and McArthur [35]. The questions include (i) “How crowded was the leisure space (selected) that you use primarily?”, and (ii) “How was the rush in the leisure space (selected) that you mainly use compared to what you expected?” Both items were rated on a five-point Likert scale. Cronbach’s α was 0.85 for the original tool, and 0.80 in the current study.

#### 2.2.2. Risk Perception

To assess risk perception, we used the four risk perception items developed by Knowles, Cutter, Walsh, and Casey [36] and modified and complemented by Hong and Cho [37]. The perception items included are as follows: (i) “I believe that it is dangerous to use the (chosen) leisure space”; (ii) “I think there is a risk of spread of COVID-19 in the (selected) leisure space”; (iii) “I think there is a high probability of making contact with a person infected with COVID-19 when visiting the (selected) leisure space”; and (iv) “I used it according to each leisure activity setting although I think there is a high risk of infection for COVID-19 when visiting the (selected) leisure activity space.” Each item was rated on a five-point Likert scale. The Cronbach’s α was 0.83 for the original tool, and 0.91 in the current study.

#### 2.2.3. Spatial Proximity

This study classified the Korean participants’ leisure activity spaces in the COVID-19 context into four zones, according to spatial proximity as proposed by Hall [38]. These were (i) “intimate space”, which is within the range of 45 cm between two persons, allowing for physical contact with outstretched arms and legs; (ii) “personal space”, which ranges between 50 cm and 120 cm, allowing for physical contact by mutual consent; (iii) “social space”, which ranges between 120 cm and 360 cm, a distance that does not allow physical contact, with the next person visible in full; and (iv) “public space”, beyond the range of 360 cm, a distance that does not allow personal interactions. The one-item questionnaire for spatial proximity was modified and complemented to suit the purpose of this study, and was rated on a five-point Likert scale.

### 2.3. Data Analysis

Frequency analysis was performed using IBM SPSS Statistics for Windows, version 25.0 (IBM, Armonk, NY, USA) to investigate the sample characteristics. In addition, exploratory factor analysis was performed to analyze the validity of the measurement tools. A one-way ANOVA was conducted to confirm the difference between the measured variables, and Schffé post-hoc tests were performed for post-hoc analysis. To increase the reliability of the analysis, we set the significance level at *p* < 0.05.

## 3. Results

### 3.1. Differences in Perceived Crowding and Risk Perception Depending on the Sociodemographic Characteristics of Korean Leisure Activity Participants

The sociodemographic characteristics of the Korean leisure activity participants are presented in Table 1. Regarding the sex-dependent differences in perceived crowding and risk perception, the mean value of perceived crowding was 2.79 (SD = 0.90) for male participants (N = 496, 46.0%) and 3.02 (SD = 0.90) for female participants (N = 582, 54.0%). The mean value of risk perception was 2.83 (SD = 0.97) for male participants and 3.08 (SD = 0.96) for female participants. The sex differences were not statistically significant. The age-dependent differences in the mean values of perceived crowding and risk perception were 2.90 (SD = 1.00) and 3.23 (SD = 0.88), respectively, for participants in their 20s (N = 262, 24.3%), and 2.65 (SD = 0.71) and 2.91 (SD = 0.90), respectively, for participants in their 50s (N = 117, 10.9%). Thus, participants in their 20s showed a significantly higher degree of risk perception than those in their 50s. The spatial proximity-dependent differences in the mean values of perceived crowding and risk perception were 2.99 (SD = 0.86) and 3.21 (SD = 0.82), respectively, in social space (N = 324, 30.1%) and 2.42 (SD = 1.12) and 2.67 (SD = 1.18), respectively, in intimate space (N = 123, 11.4%). Thus, social distance was associated with higher degrees of crowded perception and risk perception compared to intimate space, with statistical significance. Lastly, regarding the differences according to the leisure activity type, perceived crowding scored high (M = 3.47, SD = 0.70) in tourism (N = 97, 9.0%), and risk perception scored high (M = 3.59, SD = 0.65) in culture and arts (N = 82, 7.6%).

### 3.2. Sociodemographic Characteristics (N = 1078) According to Participants’ Spatial Proximity

Table 2 and Table 3 present the analysis results of the differences in perceived crowding and risk perception among participants depending on spatial proximity characteristics. Regarding “intimate space”, perceived crowding scored high (M = 3.64, SD = 0.80) in “social and other activities” (N = 7, 5.7%) and low (M = 2.02, SD = 0.92) in “relaxation” (N = 68, 55.3%). Perceived crowding and risk perception scored high (M = 3.88, SD = 0.67) in “culture and arts” (N = 6, 4.9%) and low M = 2.17 SD = 1.12, in “relaxation” (N = 68, 55.3%).

In “personal space,” perceived crowding scored high (M = 3.56, SD = 0.58) in “tourism” (N = 51, 8.9%) and low (M = 2.70, SD = 0.87) in “hobbies and entertainment” (N = 94, 16.5%). Risk perception scored high (M = 3.52, SD = 0.69) in “culture and arts” (N = 44, 7.7%) and low (M = 2.90 SD = 1.00) in “relaxation” (N = 231, 40.5%).

Lastly, differences in perceived crowding and risk perception according to the leisure activity type were as follows: perceived crowding scored high (M = 3.42, SD = 0.70) in “tourism” (N = 36, 11.1%) and low (M = 2.87, SD = 0.95) in “hobbies and entertainment” (N = 49, 15.1%). Risk perception scored high (M = 3.58, SD = 0.57) in “culture and arts” (N = 30, 9.3%) and low (M = 3.05 SD = 0.89) in “relaxation” (N = 122, 37.7%). Thus, for risk perception regarding leisure activities, significant type-dependent differences were observed in either perceived crowding or risk perception.

## 4. Discussion

This study analyzed and compared the differences in people’s perceived crowding and risk perception in leisure activity spaces. We further examined the effects on leisure activities in different spatial proximity zones, as proposed by Hall [38], and participants’ socioeconomic characteristics in the context of increasing social changes due to the COVID-19 pandemic.

First, in the analysis of age-dependent differences, participants in their 20s scored high in perceived crowding and risk perception in activity spaces, while those in their 50s scored lower. This confirms that young people are more sensitive to crowding and have a heightened awareness of the risks of COVID-19 infection [39,40,41,42,43]. Furthermore, participants in their 50s have frequently been exposed to national disaster situations, including epidemics and other emergencies, and have overcome them. This has made the older participants optimistic about the outcome of the COVID-19 pandemic. According to statistics released by the Korea Disease Control and Prevention Agency (formerly Korea Centers for Disease Control and Prevention), over half of the domestic patients infected with COVID-19 are under the age of 49. However, their mortality rate is lower than 5%, which is lower than that of the seasonal flu, while the mortality rates of patients with COVID-19 aged 50 and over rapidly increases with age [44]. Moreover, a study by Hadad et al. [45] reported that the critical effect of spatial proximity, especially among younger age groups, is characterized by a “slow evolution of significant effects and functional immaturity of the long-range orientation-specific spatial interactions, which may develop slowly, and which may be tuned by exposure to the statistics of natural scenes.” That is, the high degrees of perceived crowding and risk perception among the younger age group may be attributable to the spatial scope requiring integration, rather than to a difference in attentiveness. Therefore, measures must be taken to raise awareness of the risks of COVID-19 among people in their 50s, encourage them to avoid crowded spaces, and focus on spatial proximity to protect their health via proactive preventive measures [46]. Furthermore, more physical spaces are necessary for various leisure activities for different age groups. Thus, measures must be implemented to secure social safety distances to resolve crowding by analyzing respective activity spaces.

Second, the analysis of differences by marital status revealed no significant differences for both perceived crowding and risk perception. However, married participants had a slightly higher degree of risk perception compared to unmarried participants. This may be because unmarried people are more likely to live alone than married people, who would have inevitable contact with family members at home. This makes the unmarried participants less attentive to crowding and risk perception [47,48]. In addition, it was found that married people are more stressed and spend more time on household chores and family life than unmarried people do, yet do not fully enjoy leisure activities [49]. Thus, married people have less time to enjoy leisure activities, which is exacerbated by the warning against COVID-19 infection through mass media and social media. In effect, they may abandon leisure activities during this critical period. Therefore, it is necessary to present a variety of healthy leisure activities for married people, emphasize the importance of leisure activities, and help them to recover psychological stability.

Furthermore, according to Yang et al. [50], systematic gender differences have been observed in communicative, distributional, mobility, and spatial proximity tendencies. Women were found to be reversed in the spatial proximity context, while men tended to spend time in a narrower (and thus more predictable) range of spaces. This has the effect of structuralizing their communicative and mobility behaviors around spaces accompanied by same-gender peers.

Third, in the analysis of differences in perceived crowding and risk perception toward leisure activities depending on spatial proximity proposed by Hall [38], high degrees of perceived crowding and risk perception were associated with “social space,” that is, the person-to-person distance of 2–4 m. This suggests that the currently imposed social distancing regulations are insufficient to lower perceived crowding and risk perception [51]. Yeshurun, Yaffa, Einat Rashal et al. [52] reported that it was unclear whether the reduction of spatial uncertainty could lead to a reduction in temporal density. However, the absence of strong interactions does not negate the possibility that temporal factors may have an impact on spatial density. Additionally, the effect of temporal factors on spatial density has been demonstrated. According to the results of this study, the degrees of perceived crowding and risk perception are high at distances of 3.5 m or more, which indicates the importance of raising the current parameters for social distancing. The person-to-person safety distance during leisure activities, interpreted as the minimum safety distance regarding spatial proximity, differs from the currently set range of social distancing. It is therefore necessary to re-examine the range of social distancing, which can help reduce the crowding perception of leisure activity participants and enhance their risk perception. Additionally, the minimum safety space during leisure activities must be established and communicated via an educational warning message through social media to address people’s low risk perception tendencies.

Lastly, the analysis of the differences between leisure activity groups according to spatial proximity characteristics revealed that social and other activities were perceived as being crowded in “intimate space”, and tourism activities in personal and social spaces. This is confirmed by the fact that most of the verified cases of COVID-19 in Korea were associated with activities in intimate spaces [53]. Additionally, despite numerous COVID-19 prevention guidelines in the early phase, many people ignored or forgot the principle of safe distances and remained confused [54]. Furthermore, an increasing number of people are traveling domestically due to closed international borders, and are engaging in activities that allow them to leave their familiar spaces. Thus, people may feel crowded more acutely in a limited space, despite social distancing. Regarding risk perception by leisure activity type, while the risk of crowding was perceived more intensely in indoor settings such as culture and arts activities, movies, and museums, it was low in outdoor spaces such as parks, mountains, and beaches. Schlich, Robert, et al. [55] report that personal activities can be performed with increasing routines, but daily activities and schedules become more complicated and daily life is geared toward various pursuits and the balance of daily activities. This may be explained by the unsafe behavior arising from the belief that the risk of airborne COVID-19 infection will be low during outdoor activities, based on people’s perception that the infection rate is high in confined spaces [56,57]. However, since outdoor activities do not eliminate the risk of infection, there is a need to increase the risk perception and adhere to the given safety distances [58]. Kajosaari and Laatikainen [59] reported that indoor sports facilities were the least accessed near homes and that specialized sports facilities in larger neighborhoods with more space between dwellings were not always targeted. Considering the wide distribution of leisure activity spaces, population-level research on accessibility to sports facilities was found to be affected by larger-scale analyses [60,61] or other environments in everyday life, such as occupations and workplaces. Mackenbach, Joreintje D., et al. [62] found that that the availability of outdoor leisure activity facilities in the neighborhood was related to leisure activities, and that their selection was an important factor. This may be interpreted as individuals with high levels of leisure time tending to choose places closer to home in a specific neighborhood because of their preference for a space with leisure activity facilities. The aforementioned risk factors will likely exert a negative influence on satisfaction with leisure activities. Eventually, leisure activities must be conducted with confidence and a high-risk perception by adhering to accurate preventive guidelines. It is also necessary to ascertain the relationship between perceived crowding, risk perception, and preventive behavior. Since risk perception is a major factor with a significant impact on behavior, it should be further subdivided and investigated in future research.

## 5. Conclusions

This study empirically confirmed, using spatial proximity, the effect of leisure activity participants’ perceived crowding and risk perceptions on their leisure activities in the COVID-19 pandemic. The study results revealed that perceived crowding is not directly associated with reducing people’s participation in leisure activities. However, it has an indirect effect of decreasing participation by reducing the enjoyment of leisure activities due to fears of risk of infection through crowding.

In the COVID-19 context, it is difficult to avoid the crowding of spaces where people spend significant amounts of time due to restrictions on outside activities, with risk perception reducing participation in leisure activities. This highlights the importance of making efforts to reduce perceived crowding in leisure activity spaces. The practical implication of the finding that people perceive crowding and are aware of the presence of risk in the currently imposed social distancing is that social distancing must be reviewed to suit individual leisure activities. Each leisure activity space must implement a thorough body temperature check and safe disinfection procedure, and all leisure activity participants must always wear a mask [46]. Limiting the number of participants per hour may also be a good method to prevent crowding. More efforts must be geared toward setting strategic social distancing and path separation based on spatial proximity zones. Thus, people can gain confidence that crowding in leisure activity spaces is not directly associated with a high risk of infection and the number and array of the users of the related spaces. In addition, when leisure activity spaces safely return to their active services, leisure activity participants’ satisfaction with the adherence to safety requirements will be enhanced. As shown by the findings of this study, crowding in leisure activity spaces can reduce leisure activity participants’ interests in leisure activities due to an increase in wait time and a decrease in enjoyment due to crowding. Therefore, it is necessary to instill in leisure activity participants the belief that even a crowded leisure activity space can be a safe place for an enjoyable experience.

This study sought to identify the distance perceived to be safe for participation in leisure activities by crowding-related risk perceptions based on spatial proximity. However, it faced limitations in sampling and conducting the survey due to COVID-19 restrictions. In addition, there is no possibility of feeling more congested in sports centers or areas with a weaker distribution of cultural life. If there is only one sports center in an area, there will be more people but if there is a variety of sports centers in an area, people will be dispersed and feel less crowded. Future research should objectively categorize the degree of perceived crowding and risk perception for more accurate observations, and conduct longitudinal investigations according to various situational factors.

## Figures and Tables

**Table 1:** Differences in Perceived Crowding and Risk Perception According to Sociodemographic Characteristics (N = 1078).

Variable	N (%)	Perceived Crowding	F/t	Post Hoc Test	Risk Perception	F/t	Post Hoc Test
		M ± SD			M ± SD		
Age			2.70 *			5.13 **	20 > 40, 50
20s	262 (24.3)	2.90 ± 1.00			3.23 ± 0.88		
30s	407 (37.8)	2.86 ± 0.97			3.05 ± 0.92		
40s	292 (27.1)	2.74 ± 0.92			2.95 ± 0.97		
50s	117 (10.9)	2.65 ± 0.71			2.91 ± 0.90		
Total	1078						
Spatial proximity			15.085 ***	2, 3 > 1 4 > 2, 3		15.938 ***	2, 3 > 1 4 > 2, 3
Intimate space ^1^	123 (11.4)	2.42 ± 1.12			2.67 ± 1.18		
Personal space ^2^	570 (52.9)	2.84 ± 0.89			3.09 ± 0.88		
Social space ^3^	324 (30.1)	2.99 ± 0.86			3.21 ± 0.82		
Public space ^4^	61 (5.7)	2.43 ± 1.07			2.59 ± 1.07		
Total	1078						
Leisure activities			12.305 ***	α, β > γ		16.988 ***	α, β, γ, δ, ζ > ε α > β, δ
Culture and arts ^α^	82 (7.6)	2.84 ± 0.86			3.59 ± 0.65		
Sports ^β^	187 (17.3)	2.86 ± 0.85			3.17 ± 0.81		
Tourism ^γ^	97 (9.0)	3.47 ± 0.70			3.30 ± 0.78		
Hobbies & entertainment ^δ^	171 (15.9)	2.70 ± 0.93			3.11 ± 0.87		
Relaxation ^ε^	455 (42.2)	2.69 ± 0.99			2.79 ± 1.02		
Social activities, etc. ^ζ^	86 (8.0)	2.85 ± 0.88			3.26 ± 0.72		
Total	1078				1075		

* *p* < 0.05, ** *p* < 0.01, *** *p* < 0.001.

**Table 2:** Differences in Perceived Crowding According to the Leisure Activity Type in Different Spatial Proximity Zones (N = 1078).

	Variable	N (%)	Perceived Crowding	F/t	Post Hoc Test
Spatial proximity	Leisure activities		M ± SD		
Intimate space (0.45 m)	Culture and arts ^i^	6 (4.9)	2.83 ± 1.37	6.257 ***	ii > v vi > v
	Sports ^ii^	18 (14.6)	3.03 ± 1.02		
	Tourism ^iii^	5 (4.1)	3.20 ± 1.483		
	Hobbies and entertainment ^iv^	19 (15.4)	2.50 ± 1.13		
	Relaxation ^v^	68 (55.3)	2.02 ± 0.92		
	Social activities, etc. ^vi^	7 (5.7)	3.64 ± 0.80		
	Total	123			
Personal space (1.2 m)	Culture and arts ^i^	44 (7.7)	2.78 ± 0.86	8.401 ***	i, ii, iv, v, vi > iii
	Sports ^ii^	94 (16.5)	2.85 ± 0.81		
	Tourism ^iii^	51 (8.9)	3.56 ± 0.58		
	Hobbies and entertainment ^iv^	94 (16.5)	2.70 ± 0.87		
	Relaxation ^v^	231 (40.5)	2.73 ± 0.94		
	Social activities, etc. ^vi^	56 (9.8)	2.90 ± 0.87		
	Total	570			
Social space (3.5 m)	Culture and arts ^i^	30 (9.3)	2.95 ± 0.75	3.656 ***	vi > iii
	Sports ^ii^	67 (20.7)	2.91 ± 0.80		
	Tourism ^iii^	36 (11.1)	3.42 ± 0.70		
	Hobbies and entertainment ^iv^	49 (15.1)	2.87 ± 0.95		
	Relaxation ^v^	122 (37.7)	3.05 ± 0.89		
	Social activities, etc. ^vi^	20 (6.2)	2.50 ± 0.80		
	Total	324			
Public space (7.5 m)	Culture and arts ^i^	2 (3.3)	2.25 ± 1.77	0.768	
	Sports ^ii^	8 (13.1)	2.19 ± 1.16		
	Tourism ^iii^	5 (8.2)	3.20 ± 0.76		
	Hobbies and entertainment ^iv^	9 (14.8)	2.11 ± 0.89		
	Relaxation ^v^	34 (55.7)	2.47 ± 1.13		
	Social activities, etc. ^vi^	3 (4.9)	2.33 ± 0.58		
	Total	61			

*** *p* < 0.001.

**Table 3:** Differences in Risk Perception According to the Leisure Activity Type in Different Spatial Proximity Zones (N = 1078).

	Variable	N (%)	Risk Perception	F/t	Post Hoc Test
Spatial proximity	Leisure activities		M ± SD		
Intimate space (0.45 m)	Culture and arts ^i^	6 (4.9)	3.88 ± 0.67	8.717 ***	v > i, ii, iii
	Sports ^ii^	18 (14.6)	3.36 ± 0.77		
	Tourism ^iii^	5 (4.1)	3.85 ± 0.63		
	Hobbies and entertainment ^iv^	19 (15.4)	2.87 ± 1.14		
	Relaxation ^v^	68 (55.3)	2.17 ± 1.12		
	Social activities, etc. ^vi^	7 (5.7)	3.39 ± 0.38		
	Total	123			
Personal space (1.2 m)	Culture & arts ^i^	44 (7.7)	3.52 ± 0.69	5.860 ***	v > i
	Sports ^ii^	94 (16.5)	3.12 ± 0.71		
	Tourism ^iii^	51 (8.9)	3.28 ± 0.77		
	Hobbies and entertainment ^iv^	94 (16.5)	3.11 ± 0.81		
	Relaxation ^v^	231 (40.5)	2.90 ± 1.00		
	Social activities, etc. ^vi^	56 (9.8)	3.30 ± 0.72		
	Total	570			
Social space (3.5 m)	Culture and arts ^i^	30 (9.3)	3.58 ± 0.57	2.259 *	
	Sports ^ii^	67 (20.7)	3.21 ± 0.91		
	Tourism ^iii^	36 (11.1)	3.33 ± 0.80		
	Hobbies and entertainment ^iv^	49 (15.1)	3.27 ± 0.78		
	Relaxation ^v^	122 (37.7)	3.07 ± 0.80		
	Social activities, etc. ^vi^	20 (6.2)	3.16 ± 0.82		
	Total	324			
Public space (7.5 m)	Culture and arts ^i^	2 (3.3)	4.38 ± 0.18	2.154	
	Sports ^ii^	8 (13.1)	3.06 ± 1.08		
	Tourism ^iii^	5 (8.2)	2.70 ± 0.74		
	Hobbies and entertainment ^iv^	9 (14.8)	2.78 ± 1.21		
	Relaxation ^v^	34 (55.7)	2.31 ± 1.02		
	Social activities, etc. ^vi^	3 (4.9)	2.67 ± 1.07		
	Total	61			

* *p* < 0.05, *** *p* < 0.001.

## Data Availability

The data presented in this study are available on request from the corresponding author.

## References

[1] Kim Y.N. How to Spend the Summer Safely Amid COVID-19: 3 Dos and 3 Don’ts YHN. https://www.yna.co.kr/view/AKR20200724088800530?input=1195m.

[2] Romagosa F. (2020). The COVID-19 crisis: Opportunities for sustainable and proximity tourism. Tour. Geogr..

[3] Lee H.D. Come Play with me, Post Corona: Leisure Trend in the Corona Era. https://www.etnews.com/20200610000054.

[4] Yarımkaya E., Esentürk O.K. (2020). Promoting physical activity for children with autism spectrum disorders during Coronavirus outbreak: Benefits, strategies, and examples. Int. J. Dev. Disabil..

[5] Hoffmann B., Kobel S., Wartha O., Kettner S., Dreyhaupt J., Steinacker J.M. (2019). High sedentary time in children is not only due to screen media use: A cross-sectional study. BMC Pediatr..

[6] Bentlage E., Ammar A., How D., Ahmed M., Trabelsi K., Chtourou H., Brach M. (2020). Practical Recommendations for Maintaining Active Lifestyle during the COVID-19 Pandemic: A Systematic Literature Review. Int. J. Environ. Res. Public Health.

[7] Gyeongbuk Provincial People’s Daily Adherence to Social Distance during the May Long Weekend Has Gone Astray. http://www.hidomin.com/news/articleView.html?idxno=420756.

[8] Kim K.H. Need to Change Attitude towards Nature, Let Alone Mountain Climbing. http://san.chosun.com/site/data/html_dir/2020/05/22/2020052201471.html?utm_source=naver&utm_medium=original&utm_campaign=san.

[9] Bart N., Nijkamp P. (2012). Tourist perceived crowding and acceptability in cities: An applied modelling study on Bruges. Ann. Tour. Res..

[10] Stokols D. (1972). A social-psychological model of human crowding phenomena. J. Am. Inst. Plan..

[11] Katja G., Sattler B. (2014). Anxiety, crowding, and time pressure in public self-service technology acceptance. J. Serv. Mark..

[12] Lee N.H., Kim N.J. (2015). The Effects of Residents’ Perceived Crowding on the Emotion and Coping Behavior in Tourism Destination. Int. J. Tour. Manag. Sci..

[13] Hui M.K., Bateson J.E. (1991). Perceived control and the effects of crowding and consumer choice on the service experience. J. Consum. Res..

[14] Gifford R. (2007). Environmental Psychology: Principles and Practice.

[15] Machleit Karen A., Sevgin A.E., Susan P.M. (2000). Perceived retail crowding and shopping satisfaction: What modifies this relationship?. J. Consum. Psychol..

[16] Machleit K.A., Sevgin A.E., Susan P.M. (2001). Emotional response and shopping satisfaction: Moderating effects of shopper attributions. J. Bus. Res..

[17] Oh M.J. (2015). An Examination of the Structural Relationships between the Theme park visitors’ Perceived Crowding, Emotional Responses and Attitude. Int. J. Tour. Hosp. Res..

[18] Eom B.H., Han S.M. (1993). User’s Perception of Crowding in Lawn Areas in Park. J. Landsc. Archit. Asia.

[19] Chon T.J. (2011). The Analysis on Research Trend of Perceived Crowding in Social Carrying Capacity. J. Leis. Recreat. Stud..

[20] Belso-Martínez J.A., Mas-Tur A., Sánchez M., López-Sánchez M.J. (2020). The COVID-19 response system and collective social service provision. Strategic network dimensions and proximity considerations. Serv. Bus..

[21] Koen F., Boschma R.A., Hardeman S. (2010). Proximity and Mode 2 Knowledge Production. http://econ.geo.uu.nl/boschma/frenkenEcon&society.Pdf.

[22] Sanghoon K., Kim J., Nicholls S. (2014). National tourism policy and spatial patterns of domestic tourism in South Korea. J. Travel Res..

[23] Longley P.A., Goodchild M.F., Maguire D.J., Rhind D.W. (2005). Geographic Information Systems and Science.

[24] Yoo J.Y. (2020). Development of a New Algorithm for Avoiding Pedestrian Movement Trajectories Taking into Account Social Distancing for Social Robot. Master’s Thesis.

[25] Kennedy D.P., Gläscher J., Tyszka J.M., Adolphs R. (2009). Personal space regulation by the human amygdala. Nat. Neurosci..

[26] Leila T., Pantofaru C. (2009). Influences on Proxemic Behaviors in Human-Robot Interaction. Proceedings of the 2009 IEEE/RSJ International Conference on Intelligent Robots and Systems.

[27] Aro L., Jung-kyo L. (2019). A Study on the Personal Space Design based on Formal Psychology for Enhancing an Individual’s Sense of Stability in the Third Space. J. Korea Inst. Spat. Des..

[28] Cooper S.L. (2020). Promoting physical activity for mental well-being. ACSM Health Fit. J..

[29] Teychenne M., White R.L., Justin R., Schuh F.B., Rosenbaum S., Bennie J.A. (2020). Do we need physical activity guidelines for mental health: What does the evidence tell us?. Ment. Health Phys. Act..

[30] Choi C., Bum C.H. (2020). Changes in the Type of Sports Activity Due to COVID-19: Hypochondriasis and the Intention of Continuous Participation in Sports. Int. J. Environ. Res. Public Health.

[31] Bob J., Brenner N., Jones M. (2008). Theorizing sociospatial relations. Environ. Plan. D Soc. Space.

[32] Comrey A.L. (2013). A First Course in Factor Analysis.

[33] Heberlein T.A., Vaske J. (1977). Crowding and Visitor Conflict on the Bois Brule River.

[34] Graefe A.R., Anthony J.F. (1986). Situational and subjective determinants of satisfaction in marine recreational fishing. Leis. Sci..

[35] Hall C.M., McArthur S., Mercer D. (1994). Commercial white water rafting in Australia. New Viewpoints in Australian Outdoor Recreation Research Planning.

[36] Knowles E.S., Cutter H.S., Walsh D.H., Casey N.A. (1973). Risk-taking as a personality trait. Soc. Behav. Personal. Int. J..

[37] Hong J.Y., Cho Y.H. (2014). A Study on the Impacts of Perceived Crowding and Risk Perception on Intention to Play Ski: Focusing on the Ski Resort. J. Tour. Sci..

[38] Hall E.T. (1966). The Hidden Dimension.

[39] Fleishman L., Feitelson E., Salomon I. (2004). The role of cultural and demographic diversity in perceived crowding: Evidence from nature reserves in Israel. Tour. Anal..

[40] Golant S.M. (1983). The effects of residential and activity behavior. Elder. People Environ. Behav. Nat. Environ..

[41] Navarro J.E., Damian I.M., Fernández-Morales A. (2013). Carrying capacity model applied in coastal destinations. Ann. Tour. Res..

[42] Rasoolimanesh S.M., Jaafar M., Marzuki A., Mohamad D. (2016). How visitor and environmental characteristics influence perceived crowding. Asia Pac. J. Tour. Res..

[43] Anita Z., Raich F. (2016). The impact of perceived crowding on customer satisfaction. J. Hosp. Tour. Manag..

[44] Yoon S.Y. COVID-19, Mortality for under 40 Similar to That of Influenza; 50s and over High-Risk Group. http://dongascience.donga.com/news.php?idx=34896.

[45] Batsheva H., Maurer D., Lewis T.L. (2010). The effects of spatial proximity and collinearity on contour integration in adults and children. Vis. Res..

[46] Kim Y.J., Cho J.H., Kang S.W. (2020). Study on the Relationship between Leisure Activity Participation and Wearing a Mask among Koreans during COVID-19 Crisis: Using TPB Model. Int. J. Environ. Res. Public Health.

[47] Huang L., Qian W. (2020). An integrated model of social crowding and tourists’ environmental responsibility behaviour: Mediating effects of sense of control and self-efficacy. Curr. Issues Tour..

[48] Steen J.J.K., Iversen N.M., Hem L.E. (2019). Hotspot crowding and over-tourism: Antecedents of destination attractiveness. Ann. Tour. Res..

[49] Statistics Korea Married vs. Single—How Women’s Lives Differ?. https://www.korea.kr/news/policyNewsView.do?newsId=148714194.

[50] Yang Y., Lizardo O., Wang D., Dong Y., Striegel A.D., Hachen D., Chawla N.V. (2016). Gender differences in communication behaviors, spatial proximity patterns, and mobility habits. arXiv.

[51] Mehta V. (2020). The new spatial proximity: COVID-19, social distancing, and sociable space. J. Urban Des..

[52] Yaffa Y., Rashal E., Tkacz-Domb S. (2015). Temporal crowding and its interplay with spatial crowding. J. Vis..

[53] Kang Y.J. (2020). Characteristics of the COVID-19 outbreak in Korea from the mass infection perspective. J. Prev. Med. Public Health.

[54] Ha K. (2020). The principle of distance during COVID-19 outbreak in Korea. Int. Microbiol..

[55] Schlich R., Schönfelder S., Hanson S., Axhausen K.W. (2004). Structures of leisure travel: Temporal and spatial variability. Transp. Rev..

[56] Lidia M., Cao J. (2020). Airborne transmission of SARS-CoV-2: The world should face the reality. Environ. Int..

[57] Setti L., Passarini F., De Gennaro G., Barbieri P., Perrone M.G., Borelli M., Palmisani J., Di Gilio A., Piscitelli P., Miani A. (2020). Airborne transmission route of COVID-19: Why 2 meters/6 feet of inter-personal distance could not Be enough. Int. J. Environ. Res. Public Health.

[58] Dominski F.H., Brandt R. (2020). Do the benefits of exercise in indoor and outdoor environments during the COVID-19 pandemic outweigh the risks of infection?. Sport Sci. Health.

[59] Anna K., Laatikainen T.E. (2020). Adults’ leisure-time physical activity and the neighborhood built environment: A contextual perspective. Int. J. Health Geogr..

[60] Eime R.M., Harvey J., Charity M.J., Casey M., Westerbeek H., Payne W.R. (2017). The relationship of sport participation to provision of sports facilities and socioeconomic status: A geographical analysis. Aust. N. Z. J. Public Health.

[61] Van Tuyckom C. (2011). Macro-environmental factors associated with leisure-time physical activity: A cross-national analysis of EU countries. Scand. J. Public Health.

[62] Mackenbach J.D., de Pinho M.G.M., Faber E., den Braver N., de Groot R., Charreire H., Oppert J.M., Bardos H., Rutter H., Compernolle S. (2018). Exploring the cross-sectional association between outdoor recreational facilities and leisure-time physical activity: The role of usage and residential self-selection. Int. J. Behav. Nutr. Phys. Act..

